# Looking for new treatments of Infantile Colic

**DOI:** 10.1186/1824-7288-40-53

**Published:** 2014-06-05

**Authors:** Francesco Savino, Simone Ceratto, Angela De Marco, Luca Cordero di Montezemolo

**Affiliations:** 1 Dipartimento di Scienze della Sanità Pubblica e Pediatriche, Università di Torino, Ospedale Infantile Regina Margherita - Città della Salute e della Scienza di Torino, Turin, Italy

## Abstract

Infantile colic is a common disturbance occurring in the first three months of life. It is a benign condition and one of the main causes of pediatric consultation in the early part of life because of its great impact on family life. Some pediatricians are prone to undervalue this issue mainly because of the lack of evidence based medicine guidelines. Up to now, there is no consensus concerning management and treatment. Literature reports growing evidence about the effectiveness of dietary, pharmacological, complementary and behavioral therapies as options for the management of infantile colic. Dietary approach, usually based on the avoidance of cow’s milk proteins in breast-feeding mothers and bottle-fed infants, more recently has seen the rise of new special formulas, such as partially hydrolyzed proteins and low lactose added with prebiotics or probiotics: their efficacy needs to be further documented. Investigated pharmacological agents are Simethicone and Cimetropium Bromide: the first is able to reduce bloating while the second could reduce fussing crying, but it has been tested only for severe infantile colic. No other pain relieving agents have been proposed until now, but some clinical trials are ongoing for new drugs.

There is limited evidence supporting the use of complementary and alternative treatments (herbal supplements, manipulative approach and acupuncture) or behavioral interventions.

Recent studies have focused the role of microbiota in the pathogenesis of this disturb and so new treatments, such as probiotics, have been proposed, but only few strains have been tested.

Further investigations are needed in order to provide evidence-based guidelines.

## Introduction

Infantile colic is a common condition worldwide: about one in five infants younger than three months develops colic. Although infantile colic is considered to be a self limiting and benign affection, it is often a stressful problem for parents and a frequent and wrongly undervalued cause for pediatric consultation [1,2]. Infantile colic is defined as episodes of inconsolable crying in an otherwise healthy infant younger than three months of age, that last at least three hours a day and occur at least three days per week over the course of at least three weeks in a month, a definition first proposed by Wessel [3]. According the more recent definition by Hyman [4], colicky infants cry constantly during the evening at about the same time each day on at least one week, but they are otherwise healthy. Other signs frequently associated to inconsolable crying are flushing, abdominal distension and leg contracture. In addition, changes to the crying sounds (higher pitch), burping, needing to eat, difficulty with passing stools, tight fists, kicking, arching the back and other manifestation of pain are reported in literature. Fortunately, infantile colic is not meant to last long: it usually begins at about two weeks of age and improves by the fourth month.

### Etiopathogenesis

Despite the prevalence of the condition, the pathogenesis remains partly unknown. A theory hypnotizes that infant’s nervous or digestive system may be immature. Also behavioral issues such as family tension or inadequate interaction between parents and infant have been considered, but these issues are really controversial. Concomitant risk factors remain partially unknown; however, maternal smoking, increased maternal age and firstborn status may be associated to the development of infantile colic [5]. Infantile colic could be related to cow’s milk proteins allergy and atopy [5] (Table 1).

**Table 1:** Etiopathogenesis of infantile colic

**Main factors**	**Related factors**
Immaturity of nervous/digestive system	Family tension
Cow’s milk proteins allergy and atopy	Maternal smoking
Altered gut microflora (low Lactobacilli, increased E.coli)	Increased maternal age
Gut hormones (increased ghrelin and motilin)	Firstborn status

A correlation between colic and sleep disorders has been suggested, but recent findings show that the two disorders frequently occur in different infants [6]. There is growing evidence that the intestinal microbiota in colicky infants differs from that of healthy controls. In studies performed based on culturing approach a low amount of lactobacilli and an increased amount of coliform bacteria in the intestinal microbiota have been reported as a possible cause of gut dysmotility and increasing of gas production [7]. Recently, a study based on microarray revealed that infants with colic showed lower microbiota diversity and stability than control infants in the first weeks of life [8]. Another study suggests that Bifidobacterium and Lactobacillus may protect against crying and fussing [9].

Higher levels of ghrelin and motilin were found in infants affected by colic, even though further studies are required to clarify their role in infantile colic [10].

Calprotectin is a calcium-binding protein produced by immune system cells and so it could be used as an index of intestinal inflammation if measured in fecal samples. It is demonstrated to be useful in differentiate inflammatory bowel disease (higher level) from functional abdominal pain in school-age children [11], but its use in younger children is still to be clarified because the physiological levels of calprotectin in infants are higher than in older children [12].

Concerning infant colic, Olafsdottir didn’t observe difference between colicky and healthy infants [13] while Rhoads reported an increased value in colicky ones [14]. Further studies are need in order to clarify the values of calprotectin in colicky infant, also taking in account the type of feeding [12].

As a consequence of the lack of a complete comprehension of the causes of the condition, a wide spectrum of treatment modalities has been suggested [1,15,16].

### Diagnosis

The diagnosis is easy getting an exhaustive anamnesis and performing a correct physical examination in order to evaluating signs and symptoms [17] (Table 2). Other underlying serious diseases and feeding disorders must be excluded. A careful anamnesis has to include the relationship between infant’s behavior and the episodes of crying, time of day and duration of them. A complete physical examination should be performed by pediatrician. It is important to evaluate if the infant is correctly fed, is gaining weight, has diarrhea, fever or unusual stools. Additional signs and symptoms such as eczema or diarrhea should be elicited as these may be suggestive of a common condition such as cow’s milk proteins allergy. Also gastro-esophageal reflux or more uncommon but life-threatening causes such as bowel intussusception have to be evaluated. A negative physical examination in an infant showing paroxysmal and inconsolable crying indicate no need for biochemical and radiological examination.

**Table 2:** Differential diagnosis

**Frequent**	**Infrequent**
Infantile colic	Bowel intussusception
Otitis	Inguinal hernia
Gastro-oesophageal reflux	Fracture
Urinary tract infection	
Constipation	
Pyloric stenosis	

### Treatments

**Figure 1:**
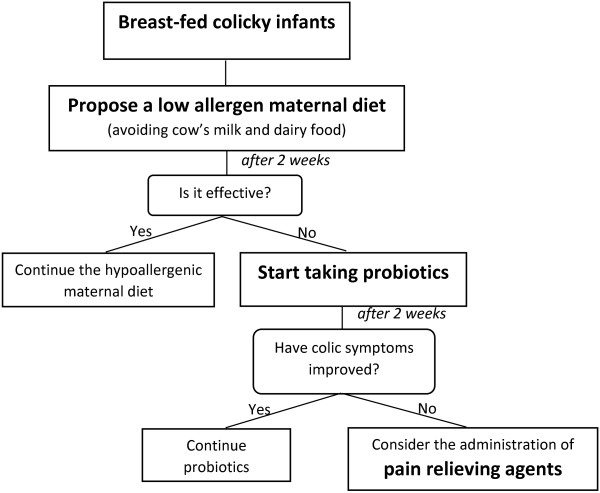
Treatment in breast-fed colicky infants.

• Dietary advices may be distinguished according the type of feeding as follow [18,19]:

▪ breast-fed infants: a monitored low allergen maternal diet avoiding cow’s milk and dairy food with appropriate intake of vitamins and minerals may be suggested. A period of at least two weeks is necessary to check the effectiveness of the diet and dietary intervention in mother has to be continued only if effective [1,20]. Evidence show that nocturnal breast milk contains melatonin, which could be useful in improving infant’s sleep and reducing colic [21] Figure 1.

**Figure 2:**
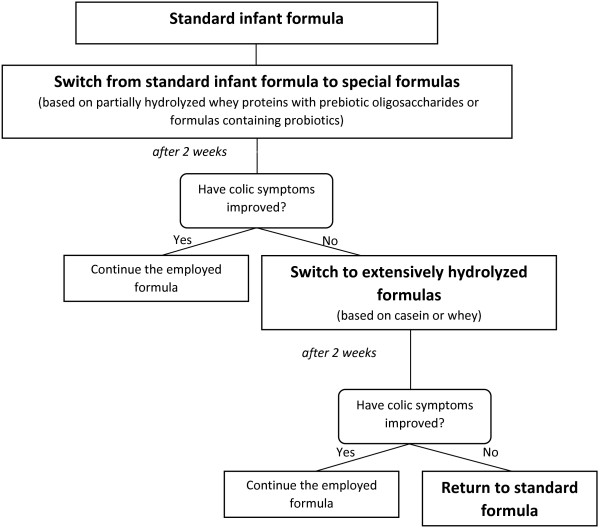
Treatment in formula fed colicky infants.

▪ bottle-fed infants: first-line approach is represented by formulas based on partially hydrolyzed whey proteins with prebiotic oligosaccharides that have been tested to be effective [22], while the efficacy of other formulas, for instance containing probiotics, need to be further documented [23]. Extensively hydrolyzed formulas based on casein or whey could be useful in children with severe infantile colic or additional atopic symptoms. However, it is crucial that any dietary changes or therapies are performed only under the supervision of the pediatrician [24] Figure 2.

• Pharmacological treatments: simethicone, which reduces gas production, may be helpful for some infants, although several randomized controlled trials noted no difference in reducing colic episodes compared with placebo [1,15]. A RCT evaluated the use of a symptomatic anticholinergic agent, cimetropium bromide, in reducing crying during colic episodes in breast-fed infants [25]. Current literature does not recommend the use of any other drugs because of reported side effects [1]. A new pharmacological agent (Nepadutant) acting on intestinal motility and sensitivity is under investigation with multi-centre, multinational, randomised, double-blind, placebo controlled study at phase IIa [26]. A Cochrane Review on pain relieving agents is in progress [27].

• Probiotics: the use of probiotics in infantile colic is based upon the hypothesis that aberrant intestinal microflora could cause gut dysfunction and gas production, contributing to symptoms. Some studies have shown that administration of *Lactobacillus reuteri* ATCC 55730 and its daughter strain *Lactobacillus reuteri* DSM 17938 to breastfed infants is well tolerated and improves symptoms of infantile colic compared with simenthicone or placebo [28-30]. The possible mechanism of action of Lactobacillus reuteri include improvement in gut function and motility as well as a possible effect on visceral pain. We could speculate that the improvement of colic effect may be related to induced changes in the fecal microbiota, since a reduction of *E. coli* colonization has been observed. At present, growing data are available on the role of probiotics in colic [31], and there is a great interest within medical research in the understanding of the mechanisms by which probiotic bacterial strains antagonize pathogenic gastrointestinal microorganisms or exert other beneficial effects in vivo [32]. Recently the use of 454-pyrosequencing analysis has been shown an increased value of Bacteroides in infants responding to probiotics [33]. A recent meta-analysis underlines that *L. reuteri* may be effective as treatment for crying in exclusively breastfed infants with colic, but there is still insufficient evidence to support probiotic use to manage colic, especially in formula-fed infants, or to prevent infant crying [23].

• Indrio et al. have performed a RCT that shows the efficacy of L. reuteri in preventing infantile colic and other functional gastrointestinal disorders [34].

• Recently, Sung et al. have described another RCT that shows no effect of L. reuteri in treating infantile colic, but this study has been conducted in a really heterogeneous population of infants with many confounding factors (such as treatment with proton pump inhibitors, types of infant formulas, recruitment at emergency department and outcomes including fussing, that is not an objective parameter). However, the meta-analysis reported by Sung in the same article confirms the positive effect of L. reuteri in reducing symptoms due to infantile colic [35].

• Complementary and Alternative Therapies: in the absence of safe and effective pharmacological interventions, complementary therapies have assumed an increasingly important role in the management of infantile colic.

▪ Herbal supplements: herbs such as fennel (*Foeniculum vulgare*), chamomile (*Matricariae recutita*) and lemon balm (*Melissa officinalis)* may help calming the infant and reducing abdominal distension [36,37]*.* However, the administration of herbal products in infants with colic raises some concerns about the potential nutritional effects (these treatments provided for a long time could lead to a decreased intake of milk), the lack of standard dosages and the possible content of sugar and alcohol. In conclusion, parents have to use them with attention and under medical control.

▪ Manipulative therapies: Cochrane Database Systematic Reviews and randomized trials published in last years focused on this kind of intervention for infantile colic. Chiropractic treatment may offer short-term relief (reduction of daily hours of crying compared with no treatment or placebo), but long-term benefits are not demonstrated. The controversial nature of these interventions, their popularity among caregivers and the presence of weak supportive evidence underline how further rigorous researches are needed [38].

▪ Acupuncture: standardized light stimulation of the acupuncture point LI4 twice a week for 3 weeks has shown reduction in the duration and intensity of crying, with no serious reported side effects [39]. However, a recent study has reported no significant efficacy of acupuncture in the treatment of infantile colic and the Authors suggest to use it only in clinical trials [40]. Future researches are needed in order to clarify this issue and to investigate the efficacy of other acupuncture points and modes of stimulation for the treatment of infantile colic.

▪ Behavioral interventions: parents’ responsiveness should be stimulated but with recommendations not to exhaust themselves and underlying that they can leave their infant with others when necessary. Many studies have proposed “infant massage”, although it does not significantly improve symptoms. A recent Cochrane Database Systematic Review acknowledges that “there is some evidence of benefits on mother–infant interaction, sleeping and crying, and on hormones influencing stress levels. Further research is needed”. A more recent study describes an approach based on regularity in infant’s daily care and feeding, accompanied by instructions to swaddle during sleep. The aim consists in helping the infant to establish a regular sleep–wake rhythm that can reduce parental distress and improve quality of interaction between parents and child [41,42].

### Conclusive remarks

The first step is to offer general advice and to provide parents of infant with infantile colic with reassurance, emphasizing the favourable and self-limiting nature of this condition. In the meantime a well-tolerated, multifactorial and personalized strategy should be adopted in order to provide a safe and effective therapeutic approach.

The available data in literature are not fully synthesized in an exhaustive systematic review: recently a Cochrane review concerning manipulative treatment has been published [38], while for other topics such as probiotics, pain-relieving agents and dietary treatment Cochrane reviews are in progress [19,27,42] (Table 3).

**Table 3:** Cochrane reviews about the treatment of infantile colic

**Title**	**Prot/Rev n**	**Authors**
Manipulative therapy for infantile colic	E0017	Dobson D, Lucassen PLBJ, Miller JJ, Vlieger AM, Prescott P, Lewith G
Pain relieving agents for infant colic^1^	K0015	Savino F, Tarasco V, Sorrenti M, Lingua C, Moja L, Ricceri F, Biagioli E
Dietary modifications for infantile colic^2^	M0015	Savino F, Tarasco V, Sorrenti M, Lingua C, Moja L, Gordon M, Biagioli E
Oral probiotics for infantile colic^1^	L0018	Praveen V, Praveen S, Deshpande G, Patole SK

^1^Protocol is available, review in progress.

^2^Cochrane authors are currently working on this review. No abstract is available at this time.

The lack of consensus about infantile colic management in medical literature, the physical and psychological impact affecting colicky infants and their parents and the evidence of controversial advice in many media outlets, such as website and magazines, make the production of further good-quality studies necessary in order to develop new and more effective treatments and to elaborate specific clinical guidelines.

Producing good-quality and comparable studies in order to obtain useful guidelines is not easy: first of all, we have to find specific, quantifiable and objective outcomes.

Studying infantile colic, Authors have mainly used subjective data such as caregivers’ observations of crying time: the use of new objective methods may help overcome these limitations.

Little is known about effective therapies for treatment of infantile colic, due to the lack of a complete understanding of the pathogenesis and the self limiting course of this disturb. So, continued efforts toward research and discovery in this field is mandatory for improving knowledge concerning oral probiotic supplementation, dietary approach and safe pain relieving agents.

## Competing interests

The authors declare that they have no competing interests.

## Authors’ contributions

FS: wrote the paper. SC: wrote the paper. ADeM: wrote the paper. LCdiM: wrote the paper. All authors read and approved the final manuscript.
